# Point-of-care method for total white cell count: an evaluation of the *HemoCue WBC* device

**DOI:** 10.1111/j.1751-553X.2008.01093.x

**Published:** 2009-12

**Authors:** A OSEI-BIMPONG, C JURY, R McLEAN, S M LEWIS

**Affiliations:** * Department of Haematology, Hammersmith Hospital, Imperial College Healthcare NHS TrustLondon, UK; † Department of Haematology, Imperial College, Hammersmith HospitalLondon, UK

**Keywords:** Point of care testing, leucocytes, WBC

## Abstract

Point-of-care testing (POCT) is becoming an important adjunct to haematology laboratory practice. An important component of the blood count is the total white cell count (WBC). Previously, this required laborious microscopic cell counting, but it can now be performed by means of automation; however, in many under-resourced countries, costly automated counters are only available in very few central hospitals. Moreover, neither method is practical in most POCT situations. The *HemoCue WBC* has been developed as a simplified alternative method, consisting of a reagent pre-loaded disposable cuvette together with basic image analysis technology. This report describes an assessment of its utility. The WBC of 500 routine blood samples from the hospital were tested in parallel by the *HemoCue WBC* and by a reference analyser to assess accuracy and utility of the former. The tests included precision, linearity, type of blood sample and anticoagulant and potential interfering substances in blood specimens. In the tests for accuracy, 192 of the 200 showed percentage difference from the NEQAS reference of <10% whilst the remaining eight samples differed by <12%, thus meeting the requirements of Clinical laboratory improvement amendments (CLIA)-88 regulations. Of the samples tested with potential interfering substances only those with >2% normoblasts or reticulocytosis showed significant differences from the reference measurements. The *HemoCue WBC* is reliable for WBC counts within the analytical range of 0.4–30.0 × 10^9^/l, except in samples where there are significant numbers of normoblasts or reticulocytes. It is simple to use and provides a valuable advance in the facilities available for POCT in haematology.

## Introduction

Point-of-care testing (POCT) is becoming an important adjunct to haematology laboratory practice. Apart from its use in hospital out-patient and intensive care/critical care departments, it provides a facility for diagnostic tests to be undertaken at primary health centre clinics or in general practice (Lewis, Osei-Bimpong & Bradshaw, 2004) and it is particularly useful when patients live a distance away from a hospital laboratory. Guidelines on the organization of POCT in haematology have been published, inter alia, by the British Committee for Standards in Haematology (England *et al.*, 1995) and the International Council for Standardization in Haematology (Briggs *et al.*, 2008). Several haematological tests are appropriate for POCT, especially measurement of haemoglobin by simple portable photometers, including those developed by HemoCue (Von Schenck, Falkebsson & Lundberg, 1985; Neville, 1987; Morris, Pont & Lewis, 2001).

Another important component of the blood count is the total white cell count (WBC). Previously, this required laborious microscopic counting of diluted blood in a counting chamber, but it can now be performed by means of an automated blood cell counter. However, in many under-resourced countries; costly automated counters are only available in very few central hospitals and other larger laboratories (Bates & Mendelow, 2006). Neither method is practical in smaller clinics, nor in most POCT situations, including general practice.

**Figure 1:**
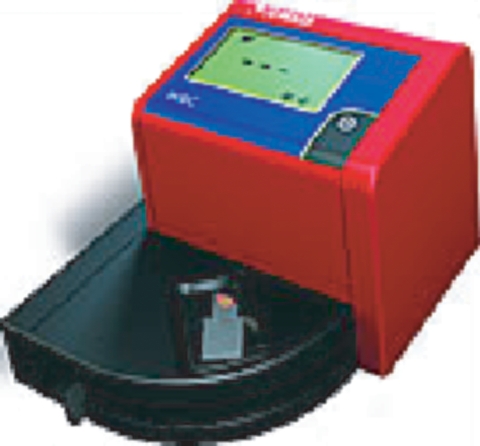
Photograph of the portable *HemoCue WBC* Point-of-care analyser (plastic cuvette containing a drop of blood is shown placed on the circular base holder).

**Figure 2:**
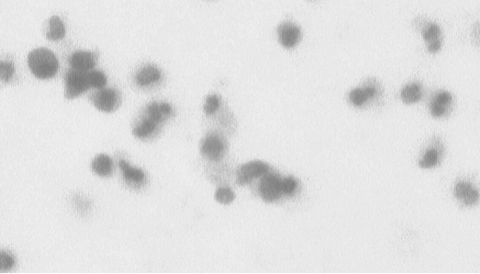
Microscopic appearance of a cuvette filled with a blood sample. (×40) Photographed on a conventional light microscope (Nikon E400; Nikon Electronic Company, Osaka, Japan).

To overcome this problem, HemoCue AB has developed a new portable system (*HemoCue WBC*, Figure 1). It consists of a microscopic image detector (photomicroscope), a cuvette holder and an LCD display unit; it is powered by six AA batteries or an AC 6 volt adapter, and is as simple to use as the *haemoglobinometers*. Approximately 10 μl of peripheral capillary blood or venous blood in any anticoagulant is drawn into a plastic cuvette containing a reagent where the red cells are haemolysed and the nuclei of the white cells stained by methylene blue. In the analyser, an image is captured by the photomicroscope (Figure 2) and after 2 min, the image analysis programme counts the stained white cell nuclei, ‘gating out’ platelets that are much smaller than white cells. The WBC is then expressed on the LCD as the WBC × 10^9^/l.

The instrument is designed to measure WBC counts between 0.4 and 35.0 × 10^9^/l, beyond this limit, a flag is triggered; an LLL flag appears on the display to denote a count below its counting capability when the WBC is <0.4 × 10^9^/l and an HHH flag is triggered when the count is higher than 35.0 × 10^9^/l to indicate a WBC count above its counting capability.

The instructions are easy to follow and after a short ‘hands-on’ trial the instrument could be used without any problems even by persons with no previous experience in laboratory technology.

This study was undertaken to assess the reliability and clinical utility of the device in accordance with the recommendations of the International Council for Standardization in Haematology (1994) and taking account of the requirements established by UK NEQAS that for the WBC counts to be acceptable the results must be within 8–10% of assigned values (Lewis & De la Salle, 2006) as well as the Clinical laboratory improvement amendments (CLIA)-88 requirement that results within 15% of assigned value are clinically acceptable (US Federal register, 1992). Accordingly, in the present study assessments were made at these cut-off points.

Whilst this device is intended primarily for use with capillary blood, for the purpose of this study, it was logistically more practical to use venous blood samples collected into EDTA. A previous study showed no significant differences in the WBC between venous and capillary blood (Yang *et al.*, 2001) and the International Council for Standardization in Haematology and International Society for Hematology have also established that there are negligible differences in blood count parameters between venous blood and capillary blood provided that the capillary sample is obtained from free flow of the blood without squeezing, and after discarding of the first drop (Tatsumi *et al.*, 2002). Although this is not strictly relevant to an assessment of the ability of the *HemoCue WBC* to measure the WBC in the sample presented to it, in order to provide assurance on its utility for measuring capillary blood in practice, a comparison of results between the two procedures was assessed on a limited number of volunteer laboratory staff.

## Methods

The study assessed the manufacturer’s claims that accuracy and linearity are within 6% of the true values in the range 0.3–35 × 10^9^/l. The evaluation included tests for precision, comparability, accuracy, stability, and any interference to the WBC by various factors such as nucleated red cells, and disease states such as leukaemia, lymphoma and iron deficiency. In addition to routine specimen collection in dipotassium–EDTA, 50 specimens were also collected in tripotassium–EDTA and in sodium citrate to assess any significant differences because of the anticoagulant.

Samples were obtained from 500 blood specimens in dipotassium–EDTA anticoagulant that had been sent to the laboratory for routine blood counts. Reference counts were obtained by a standardized Sysmex XE-2100 analyser (Sysmex Corporation, Kobe, Japan). Comparisons between *HemoCue WBC* and the reference analyser were assessed in several groups, namely WBC below normal, within normal range, above normal range, and at borderline between normal and abnormal. To take account of the limits of acceptable performance established by NEQAS and by CLIA-88 (International Council for Standardization in Haematology, 1994; Morris, Pont & Lewis, 2001), special note was made of any differences from the reference at 6%, 8–10% and 15%, respectively.

To assess the effect of sample type, WBC measurements were made on blood from eight laboratory staff volunteers from whom the samples were obtained by finger prick in parallel with venous blood collected in EDTA.

Linearity studies were carried out by serially diluting a blood specimen with a WBC of 30.0 × 10^9^/l one in two up to one in 16 volumes of isotonic saline. The WBC was then performed on each sample by the *HemoCue WBC* and by the reference analyser.

The flagging capability of the *HemoCue WBC* was also assessed; the trigger factors and their sensitivity levels were noted during the evaluation of accuracy and linearity.

The effect of cuvette storage temperature on the WBC results was assessed by testing samples with cuvettes stored at 4 °C, room temperature (22 °C) and 37 °C.

### Statistical methods

All the data were analysed using Excel software statistics package analysis software (microsoft office excel 2003; Microsoft Corporation, Redmond, WA, USA); the mean, range and student paired *t-*tests were calculated using this package; *P-*values < 0.05 were considered significant.

## Results

### Precision

Mean values, ranges, standard deviation and CVs were established on five replicate tests at eight different levels of WBC (Table 1).

**Table 1:** Precision data showing degree of variation of *HemoCue WBC* values over a range of white blood cell counts

Replicate mean WBC × 10^9^/l (*n* = 5)	WBC range × 10^9^/l	CV (%)	SD × 10^9^/l
0.7	0.5–0.8	11	0.08
1.5	1.4–1.7	8.6	0.13
3.7	3.4–3.9	3.0	0.11
5.5	5.0–6.1	4.5	0.25
8.5	8.2–8.8	2.2	0.19
14.3	13.1–15.2	3.3	0.47
21.2	20.6–21.9	2.8	0.59
27.4	26.0–28.4	2.0	0.54

### Comparability

**Figure 3:**
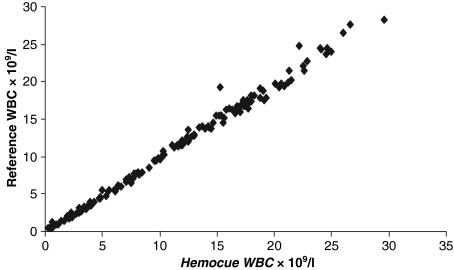
Correlation between the reference analyser and the *HemoCue WBC*. *Y* = 0.989*X*−0.082 (*Y* = reference analyser and *X* = *HemoCue WBC*). The correlation coefficient (*r*) between 0.4 × 10^9^/l and 30 × 10^9^/l = 0.997. These findings indicate good comparability within the manufacturer’s suggested analytic range with no detectable bias.

White cell counts on 200 samples were performed on the *HemoCue WBC* and the reference analyser; the data were assessed for comparability which is graphically represented in Figure 3.

### Comparison of capillary and venous blood samples

**Figure 4:**
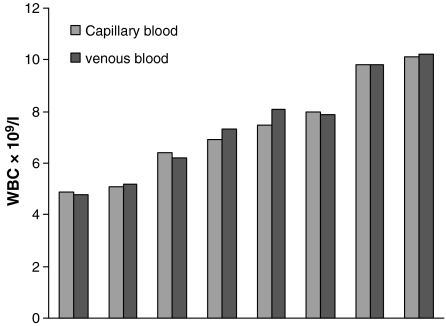
Comparison of *HemoCue WBC* measurement*s* on capillary and venous blood samples:

White cell counts were performed with the *HemoCue WBC* on blood from eight volunteers, comparing samples obtained by finger prick to those from venous blood. Paired results are shown in Figure 4. Measurements did not vary by >5% in any case and no bias was detected. It was concluded that there was no significant difference between the two methods of specimen collection (*P*=0.105).

These findings indicate good linearity within the manufacturer’s suggested analytic range with no detectable bias. There was no significant difference in the linearity of the *HemoCue WBC* in comparison to the reference analyser (*P* = 0.475; Table 2).

**Table 2:** Linearity serial dilutions of a sample with initial WBC = 30.0 × 10^9^/l

Dilution	Reference analyser count × 10^9^/l	*HemoCue WBC* count × 10^9^/l
Neat	30.0	29.8
1:2	14.9	14.5
1:4	10.6	10.0
1:8	5.8	6.0
1:16	3.4	3.1

### Accuracy

The accuracy of the *HemoCue WBC* was assessed by 500 counts at various ranges in comparison with the reference analyser. The numbers of paired results within and outside clinically acceptable limits (see above) are shown in Table 3.

**Table 3:** Accuracy showing percentage difference of *HemoCue WBC* values from reference count

WBC range × 10^9^/l	Number of samples analysed	<10% difference	>10% < 15% difference	>15% difference
0.1*–4.0	110	107	3	0
4.1–10.0	88	71	17	0
>10.0–12.0	109	108	1	0
>12.0	93	91	2	0
>12–15	38	37	1	0
>15–20	34	33	1	0
>20–30**	28	27	1	0

* Samples with the reference WBC <0.4 were flagged as low with an error code: LLL.

** Samples with the reference WBC >30 were flagged as high with an error code: HHH.

Only three of the 110 samples tested with WBC values below the normal range showed a variation of >10% from the reference counter; however, in these three cases, the variation was <12%. In the group with counts within the normal range, 71 samples correlated to the reference count to within a 10% difference, whilst 17 were within the clinically acceptable level of a 15% difference. In the group with counts above the normal range, 98% of counts were within a 10% difference whilst the remainder fell within a difference of 15%.

The accuracy studies also included measurement on two NEQAS survey samples. The first sample gave a *HemoCue WBC* count of 0.9 × 10^9^/l, as compared with the participants all-methods mean (*n* = 1500) of 1 × 10^9^/l. On the second sample, the *HemoCue WBC* count was 3.9 × 10^9^/l whilst the participant all-methods mean was 4.0 × 10^9^/l. These results gave a highly satisfactory performance by the *HemoCue WBC* with deviation index scores of <0.5 for both samples.

### Stability

The mean and median of the WBC obtained using cuvettes equilibrated to three different temperatures are shown in Table 4.

**Table 4:** Comparison of *HemoCue WBC* values against reference analyser at different temperatures

	Reference WBC × 10^9^/l	4 °C *HemoCue WBC* × 10^9^/l, *P =*0.437*	22 °C *HemoCue WBC* × 10^9^/l, *P*=0.525*	37 °C *HemoCue WBC* × 10^9^/l, *P*=0.234*
Range	1.6–19.5	1.7–18.9	1.5–19.6	1.5–19.3
Mean	10.7	10.7	10.6	10.7
Median	10.4	10.3	10.1	10.7

* *P*-value is shown for comparison between *HemoCue WBC* and corresponding reference WBC.

There was no statistical difference between the *HemoCue WBC* results and the reference values at the three temperatures, indicating that *HemoCue WBC* counts showed no variation with alteration to the recommended cuvette storage temperature.

### Anticoagulants

Effects of the type of anticoagulant on the count were evaluated on samples that had been collected in sodium citrate and tripotassium–EDTA anticoagulants as well as in the recommended dipotassium–EDTA. There were no significant differences in the counts from different specimen containers and results showed a good correlation with the reference counts (Table 5).

**Table 5:** Comparison of *HemoCue WBC* values using different anticoagulants

	*n*	Citrate reference	Citrate *HemoCue P*=0.426	K_2_EDTA reference	K_2_EDTA *HemoCue P*=0.359	K_3_EDTA reference	K_3_EDTA *HemoCue P =*0.525
Mean WBC × 10^9^/l	50	9.7	9.8	9.9	9.9	9.8	9.9
Median WBC × 10^9^/l	50	9.8	9.7	9.8	9.8	9.9	9.8

*P-value is shown for comparison between *HemoCue WBC* and corresponding reference WBC.

### Interference

The possibility was assessed of interference with the WBC by the presence of other types of cells (reticulocytes, nucleated red cells) thrombocytosis and certain other abnormal blood conditions, as listed in Table 6.

**Table 6:** Comparison of WBC values for various conditions and interference

Disease/condition	*n*	*HemoCue WBC* mean (×10^9^/l)	Reference count mean(×10^9^/l)	*P-*value
Sickle cell disease	30	10.6	9.2	<0.001
Iron deficiency anaemia	35	9.8	9.7	0.134
Acute leukaemia	35	35.1	30.0	<0.001
Chronic leukaemia	32	40.0	36.0	<0.001
Lymphoma	30	20.0	19.6	0.187
Myeloma	31	15.6	15.3	0.216
Reticulocytosis (>100 × 10^9^/l)	25	10.8	9.2	<0.001
Thalassemia with nucleated red cells	30	11.2	9.8	<0.001
Thrombocytosis	35	9.1	9.2	0.175

The mean *HemoCue WBC* count was significantly higher than the mean reference count (*P*<0.001) in patients with sickle cell disease or thalassaemia major with significant numbers of normoblasts (i.e. >2%). The *HemoCue WBC* mean count was also significantly higher in samples where there was a reticulocytosis: >100 × 10^9^/l as counted by the reference analyser and subsequently confirmed by blood film morphology. There were no flags generated by the analyser to alert the user to these discrepant results.

However, the platelet count, even at the high levels in thrombocytosis (1000 × 10^9^/l), had no influence on the *HemoCue WBC* results (*P*=0.17), and reliable WBC counts were also obtained in iron deficiency, lymphoma and myeloma.

## Discussion

There has been a long-felt need for a method to obtain white blood cell counts at point-of-care that could be as useful for patient management as the well-established methods for haemoglobinometry. It is essential that such a device should be simple to operate, unaffected by various climatic conditions and sufficiently accurate for reliable clinical use.

Our study has indicated that these requirements are provided by the *HemoCue WBC* system as described above. This portable device is simple to use, even by persons with no previous experience in laboratory technology. Although the *HemoCue WBC* does not perform a differential count, a basic WBC (especially when linked to haemoglobin) will often help expedite clinical management. Furthermore, as primary healthcare units in under-resourced rural areas often have the facility of microscopy (Bates & Mendelow, 2006), an abnormal WBC would be an indication for examination of a blood film and a differential leucocyte count.

The extensive assessment of the utility and reliability of the *HemoCue WBC* described in this paper has shown good precision for replicate measurements on blood samples over a range of WBC counts and good comparability with results obtained by more sophisticated blood count analysers. For assessment of accuracy, results were compared with those measured by a calibrated reference blood cell analyser: 94.8% of the samples tested were within the acceptable performance limits of 8–10% of the correct measurements as required by UK NEQAS (Lewis & De la Salle, 2006), and in no case was the difference >12%, so that all were acceptable in accordance with the CLIA-88 requirement that results should be within 15% (US Federal register, 1992). Thus, the small differences from the reference measurement would not be clinically misleading and especially when results are considered in absolute numbers rather than percentages. Furthermore, tests on two samples in UK NEQAS surveys gave results very close to the overall mean values for these surveys.

In our study, the device provided reliable comparability in the range of 0.4–30.0 × 10^9^/l. This is slightly narrower than the range of 0.3–35 × 10^9^/l specified by the manufacturer; however, samples with WBC < 0.4 × 10^9^/l were flagged as low (code LLL) and conversely, those with counts above 30.0 × 10^9^/l were flagged as high (code HHH); thus, in a clinical setting, this flagging would provide adequately reliable information.

Special note should be taken of the cases recorded in Table 6 where measurements may be distorted by specific interfering substances, notably reticulocytosis and more than 2% circulating normoblasts. Haemoglobinopathies (especially sickle cell disease and thalassaemia major) were identified as the main cause of falsely high counts, because of two factors: (i) the image analysis programme does not distinguish nucleated red cells from the total WBC, and (ii) as reticulocytes have a greater resistance to lysis than mature red cells (Sadallah, Hano & Schiferlli, 2007). The 2-min reaction of the sample with the lytic reagent in the cuvette may not be adequate in these circumstances. The restriction of the flagging capability of the *Hemocue WBC* analyser to extreme WBCs (<0.4 and >30.0 × 10^9^/l) is a major limitation of the device; no flags are generated to alert the user to increased numbers of reticulocytes and normoblasts. Therefore, in view of the discrepancies in the white blood counts which were found in blood samples of patients with sickle cell disease and thalassaemia, care must be taken in interpreting the *HemoCue WBC* measurements in regions where these conditions are prevalent. However, even there, the method provides a clinically useful approximation of the WBC.

In a limited study, the sample type (venous or capillary blood) did not make any difference to the WBC. Stability studies were also carried out to assess the effects of different local practice and climatic conditions. It was shown that tests were not affected when the cuvettes were stored at temperatures between 4 and 37 °C; nor were they influenced by different anticoagulants.

## Conclusion

The total white cell count (WBC) was recognized as an important test for health screening and for diagnosis and clinical management of patients. This study has demonstrated that the newly developed *HemoCue WBC* provides a simple method to obtain reliable measurements with an accuracy that is comparable with that of a standardized reference analyser. It is, thus, eminently suitable for use in any point-of-care situations with limited or no laboratory facilities, especially in general practice and in rural clinics without access to modern blood count analysers provided that the users appreciate that in patients with sickle cell anaemia or thalassaemia, and also when there are significant numbers of reticulocytes or normoblasts in circulation, the WBC may give an inaccurate reading. However, even so, the method will provide a clinically useful approximation of the WBC.
